# Supplementary material for: Are carbon emissions trading and green financial instruments synergistic? -Comprehensive quantitative research based on content analysis
Source: PLoS One. 2024 Mar 7;19(3):e0298601. doi: 10.1371/journal.pone.0298601 (PMC10919601; doi:10.1371/journal.pone.0298601)
Supplement: S1 Data — (ZIP) [file pone.0298601.s001.zip › Data/地级市Z_F工作报告.zip!/╔╜╢1⁄2/╡┬╓▌╩╨/└┤╫╘╣1⁄2╓┌║┼í╛┬φ┐╦╩2╛▌═°í┐_2019.docx]

各位代表：

现在，我代表市人民政府向大会报告工作，请予审议，并请市政协委员和列席会议的同志提出意见。

一、2018年主要工作情况

2018年，在中共德州市委的坚强领导下，在市人大、市政协的监督支持下，我们坚持以习近平新时代中国特色社会主义思想为指导，深入学习贯彻党的十九大精神，全面贯彻落实习近平总书记视察山东重要讲话、重要指示批示精神，坚持稳中求进工作总基调，积极践行新发展理念，认真落实高质量发展要求，加快新旧动能转换，围绕实施协同发展战略、建设协同发展示范区工作主题，从严从实、奋发有为，经济社会保持持续健康发展。全年实现地区生产总值3380亿元，增长6.7%，增幅居全省第7位；一般公共预算收入202.5亿元，增长8%，增幅居全省第6位，税收占比76.8%，较上年提高5.7个百分点；固定资产投资增长7.3%，增幅居全省第6位，“四新”经济投资占固定资产投资比重达到44%；规模以上工业增加值、主营业务收入、利润分别增长7.3%、7.2%、15.9%；工业税收增长11.1%，连续24个月保持两位数增长；社会消费品零售总额1525.5亿元，增长9.3%；城镇和农村居民人均可支配收入分别增长7.8%和8.8%。县域经济发展质量不断提高，齐河、禹城再次跻身全国百强县。

（一）协同发展迈出新步伐

认真落实省委省政府《关于支持德州加快融入京津冀协同发展的意见》，抓住用好京津冀协同发展战略机遇，推行“1+1+6”工作机制，统筹规划，主动对接，“一区四基地”建设取得积极成效。全年新开工京津冀项目124个，到位资金115.7亿元。北京有研科技大尺寸硅材料规模化生产基地、高新区新能源装备产业园、中建材凯盛晶华绿色建材产业园等一批大项目好项目相继签约或开工建设。“研发在京津、转化在德州”效应不断放大，1100余家企业与京津地区600多家高校科研机构建立稳定合作关系。中国教科院、北师大、北京新学校研究院等优质教育资源和北京同仁医院、阜外医院、天津医科大学等高水平医疗机构来德合作。积极融入省会、服务济南，基础设施对接，产业发展协作，优势资源共享，齐河黄河公路大桥建成通车，济青高铁南联络线及齐河站建成运营，与省会交通连接更加便捷，交流合作更加密切。

（二）高质量发展呈现新态势

全面展开新旧动能转换重大工程。全市储备项目500个、投资规模5300亿元，40个项目列入省新旧动能转换重大项目库。新能源汽车及零部件、生物技术、医养健康、现代农业4个产业纳入全省首批现代优势产业集群。新认定省级高新技术企业87家、科技创新平台61家，转化科技成果220项，高新技术产业总产值占规模以上工业总产值的比重达到38%。 121个市级重点项目完成投资519.7亿元，150个工业重点技改项目顺利实施，年度完成投资195.3亿元。深入实施“三航”计划，恒源石化、永锋集团首次进入“中国企业500强”；新增报辅导企业2家、挂牌企业6家，完成规范化改制440家，全市实现直接融资301.3亿元。常态化开展“进解促”工作，在全省率先开展无还本续贷，累计为1227家企业发放无还本续贷1957笔、130.8亿元。5个县级化工产业园获批，7家城镇人口密集区危化品生产企业启动搬迁改造，294个化工项目完成整改。全市清理低效土地1.5万亩，六大高耗能行业增速全线回落，万元GDP能耗超额完成省定目标。坚持“用效益体现价值、用财富回报才智”，成功举办“智汇德州”人才创新创业周，自主培养省级以上高层次人才117人，柔性引进人才1285人，建成人才公寓11161套，累计为925名重点高校毕业生发放生活补贴2300余万元，人才政策“黄金30条”效应持续放大。

扎实推进乡村振兴。按照省委“德州要在打造乡村振兴齐鲁样板中率先突破”的要求，出台乡村振兴五年规划和五个专项方案，制定打造乡村振兴齐鲁样板率先突破三年行动方案，乡村振兴战略顺利开局。传统农业转型提升，全年粮食总产177.6亿斤，新评选认定“放心农场”44家，新增设施蔬菜6.2万亩、青贮玉米18.1万亩，建设标准化规模养殖场（区）258处，“三品一标”认证产品达到453个、面积762万亩，农产品抽检合格率98%以上。现代农业加快培育，农业集约化水平不断提高。规模以上农产品加工企业增至861家，土地流转面积占比达到45%，发展农民合作社1.6万家、家庭农场3044家，农村党支部领办创办土地股份合作社扎实推进，供销为农服务中心等各类新型农业服务组织快速发展，凯盛浩丰、水发集团、新希望六和等124家田园综合体和现代农业产业园区正在成为德州农业开放合作、转型发展的新引擎。美丽乡村建设成效明显，全面启动农村人居环境整治三年行动，扎实推进农村“七改”，完成改厕11.7万户，新建改建农村公路1410公里。开工重点水利工程34项，新增水肥一体化面积8万亩。落实最严格耕地保护制度，“大棚房”问题清理整治行动取得阶段性成果。

加快城市建设步伐。以生态修复、城市修补为导向，推动城市总体规划修编和主城区控规全覆盖。围绕全面提升城市功能，重点实施5大类81个公共城建项目，完成投资114亿元。以“宜居宜业的现代化新城”为目标，有序推进东部城区建设，德州太阳能小镇、东部医疗中心、养老示范城、高级师范学校等一批功能性项目和中法体育器材研发生产基地、绿地创新谷等产业项目进展顺利。以完善设施、宜居便民为重点，改造提升中部城区功能，集中实施了岔河生态景观提升、雨污分流、污水处理厂升级、棚户区和老旧小区改造等一批重点工程。西部城区生态修复加快推进，大运河万亩生态林场、西陈沟和东八里湿地生态效益显现。加快城镇开发建设，10个县城拆迁改造面积376万平方米，17个国家、省、市特色小镇和重点示范镇完成投资109亿元。稳步推进重大基础设施建设，德上线京台高速至G105段和陵城、乐陵、庆云绕城国省道改造顺利实施，中心城区城市轻轨线网规划论证草案编制完成。

（三）开放合作开创新局面

高质量开展“双招双引”， 成功举办第三届京津冀鲁资本技术交易大会、青年企业家创新发展国际峰会2018助推德州新旧动能转换论坛、“儒商家园·尚德之州”双招双引恳谈会、德州优质农产品北京展示交易会等活动，全年招商引资到位资金214.8亿元。全面拓展对外开放，实现进出口292.8亿元，增长19.3%；实际利用外资2.1亿美元，新备案外资企业29家。积极融入“一带一路”建设，与沿线国家有进出口实绩企业达到643家。全市近400家企业与东盟国家开展经贸合作，进出口额达到80.6亿元。基础设施国际化互联互通取得新进展，平原到青岛港海铁联运货运量稳步增长，齐河铁路物流基地海铁联运班列开通，建立公共海外仓6个。拓宽与非洲、拉美、北欧国家的友好交流渠道，新缔结6个友好城市。成功举办第二届国际太阳能十项全能竞赛，有力提升了德州对外开放形象。

（四）三大攻坚战取得新进展

生态环境质量持续改善。深入贯彻习近平生态文明思想，认真抓好中央和省环保督察问题整改，坚持科学治污，实施精准治理，环境空气质量综合指数、PM2.5浓度分别改善12.4%、15.2%。全面启动“四减四增”行动，能源结构继续优化，煤炭消费持续下降，累计淘汰10蒸吨及以下燃煤锅炉3690台，农村地区完成清洁取暖改造52967户，城市（县城）新增清洁取暖面积597万平方米，推广洁净型煤51.2万吨。不断加大水生态修复力度，完善“河长+湖长”双保障体系，整治城市黑臭水体24条，封停地下水井1055眼，新增湿地3400亩，重点河流水质稳定达标，饮用水水源地保护工作取得扎实成效，成功创建省级节水型城市。持续实施“绿满德州·生态美市”行动，全年新增造林21.6万亩。

脱贫攻坚任务基本完成。认真落实省委省政府“八个精准”要求，扎实推进精准脱贫，累计脱贫100405人，755个贫困村全部摘帽。深化产业扶贫行动，累计实施产业项目1321个，投入专项资金9.8亿元，发放贷款9.1亿元，项目收益1.4亿元。统配行业扶贫政策，累计资助学生9626人，救治患病贫困人口21127名，改造危房4811户，免费安装有线电视2.8万户。贫困村“五通十有”基本达标，建设改造贫困村公路793公里，村村实现改水、改电。村级扶贫专岗、盘活沉睡资源等工作在全国、全省创出了经验。

重大风险得到有效防控。大力创新完善金融工具，科学推动企业破圈断链，积极防范法人机构风险，严厉打击恶意逃废金融债务、非法集资等行为，累计化解不良贷款62.8亿元，金融风险总体可控。全力防范处置政府债务风险，在全省率先把政府债务全部置换为政府债券。持续开展“大快严全”集中行动，安全生产形势总体平稳。城市违法建设治理和高铁沿线综合整治工作取得明显成效。严防严管严控食品药品安全风险，成功创建省级食品安全城市。社会治安防控体系逐步完善，扎实开展扫黑除恶专项斗争，重拳打击人民群众反映强烈、深恶痛绝的黑恶势力犯罪。积极妥善化解信访矛盾纠纷，人民群众安全感满意度持续提升。

（五）营商环境又有新改善

深化“放管服”改革。全面推进“一次办好”，以“一次办结、群众满意”为标准，推动政务服务理念、制度、作风全方位深层次转变。大力实施政务服务标准化等五项工程，开展优化营商环境十个专项行动，“一次办好”事项实现全覆盖，“3545”专项改革目标基本实现。加强政务服务大厅、便民服务中心规范化建设，在市县推行“无差别受理”改革，建立“前台综合受理、后台分类审批、统一窗口出件”的政务服务新模式。积极推动“证照分离”“多证合一”等商事制度改革，大力推行工商登记全程电子化，加强“双随机一公开”监管，全年新登记市场主体6.1万户，市场活力进一步激发。

认真做好各项重点领域改革。扎实推进新型城镇化、现代农业示范区、农村土地制度改革等23项国家级试点和鲁北人才改革试验区、产城融合推进就地城镇化等31项省级试点。落实市级机构改革实施意见，优化政府机构设置和职能配置，人员转隶、集中办公等工作有序进行。分类推进事业单位改革，86家从事生产经营活动事业单位完成改革，为乡镇卫生院、社区卫生服务中心重新核编，探索公办幼儿园、中职院校人员控制总量备案改革。深化国企国资改革，完成38家国有企业资产重组、优化整合和划转调整，实现市属经营性国有资产集中统一监管。

（六）民生保障得到新提高

集中力量实施“全面改薄”和解决城镇中小学大班额，累计投资138.4亿元，新招聘教师2016名，城镇学校大班额问题有效缓解，“全面改薄”任务超额完成。高中和职业教育水平不断提高。医疗卫生领域补短板取得积极进展，市人民医院门诊楼、市妇女儿童医院及市中医院病房楼和33个县级项目加快推进，40家公立医院综合改革全部启动，全年为群众节省药费4.9亿元，看病难看病贵问题进一步缓解。中心城区82个老旧小区改造项目全面开工，累计改造163个，总投资8.3亿元，改造面积230万平方米，近10万居民受益。扎实开展环境卫生综合整治，顺利通过国家卫生城市复审。落实就业优先战略，全市新增就业6.3万人，高校毕业生总体就业率达到95%。健全社会保障体系，企业养老保险待遇实现“十四连涨”，居民基础养老金实现“六连涨”，社会救助直接惠及城乡低保、特困人员及孤困儿童73702人。加快发展养老事业，新增养老床位4500张。扎实做好全国双拥模范城（县）创建工作，积极稳妥做好退役士兵就业安置。加强住房保障，开工棚户区改造安置住房61161套。深化文明城市创建，倡树社会主义核心价值观，持续深化“我文明德州文明”主题实践活动。公共文化服务体系更加健全，基层综合性文化服务中心覆盖率99%，1000余场文化活动、4997场送戏下乡惠及基层群众。大运河世界文化遗产公园加快建设，苏禄文化博物馆获批中国华侨国际文化交流基地。 10个县级广播电视台全部通过省一级台考核验收。竞技体育取得好成绩，亚运会金牌实现零的突破。认真落实全民健身计划，基本实现农村社区健身场地设施全覆盖。国防动员、人民防空、民兵预备役工作取得新成效，民族宗教、外事侨务、对台工作进一步加强，妇女、儿童、青少年、老年人和残疾人权益得到较好保障，海关、口岸、档案、史志、仲裁、气象、防震减灾、慈善、红十字会等工作深入开展。

（七）政府行政效能实现新提升

坚持以党的建设统领政府工作全局，认真落实全面从严治党主体责任，扎实推进“两学一做”学习教育常态化制度化，持续深化“大学习、大调研、大改进”，全面强化思想政治保障。自觉接受市人大、市政协和社会各界监督，全年办理人大代表建议414件、政协委员提案559件，12345热线平台推动解决市民诉求85.3万件。坚持依法全面履职，自觉按法定权限、规则、程序办事，清理政府规章和规范性文件188件，受理行政复议案件509件。严格落实中央八项规定及其实施细则精神，持之以恒纠正“四风”，加强对财政资金使用、公共资源交易、国有资产经营等领域监管，深入推进政府系统廉政建设和反腐败斗争，政府自身建设和治理能力不断提升。

各位代表，过去一年的成绩来之不易，值得倍加珍惜。这是全市上下全面贯彻落实党中央、国务院和省委、省政府决策部署的结果，是市委正确决策、坚强领导的结果，是市人大、市政协和社会各界有效监督、大力支持的结果，是全市干部群众团结一心、奋力拼搏的结果！在此，我代表市政府，向全市人民，向各位人大代表、政协委员、离退休老同志，向各民主党派、工商联、无党派人士、各人民团体和社会各界人士，向驻德部队官兵，向中央、省驻德单位，向所有关心支持德州发展的海内外朋友，表示衷心的感谢和崇高的敬意！

我们清醒认识到，全市经济社会发展不平衡不充分的问题仍然突出。发展质量不高、创新能力不强，实体经济发展面临不少困难；民生欠账还比较多，教育、医疗、养老、生态保护、安全生产等领域存在不少短板；财政收支矛盾突出，重点民生事业等刚性支出压力较大；营商环境不够优化，政府工作人员的法治思维、服务意识、专业素养、行政效能需要进一步提升；不正之风和腐败问题还时有发生等等。对此，我们要坚持问题导向，加强整改落实，努力在实践中不断改进。

二、2019年重点工作任务

今年，是新中国成立70周年，是全面建成小康社会关键之年，也是我市加快新旧动能转换、实现高质量发展的重要一年。当前，德州正处于经济社会发展的重要历史节点，正处于爬坡过坎、滚石上山的关键期，正处于抢抓机遇、奋力赶超的窗口期，挑战与机遇并存，只要我们坚定信心、勇于担当，迎难而上、开拓进取，德州发展就大有可为、我们的事业就大有作为。

政府工作的总体要求是：以习近平新时代中国特色社会主义思想为指导，认真贯彻党的十九大和十九届二中、三中全会精神，深入落实习近平总书记视察山东重要讲话、重要指示批示精神，按照省委省政府决策部署，在市委的坚强领导下，坚持稳中求进工作总基调，坚持新发展理念，坚持推动高质量发展，坚持以供给侧结构性改革为主线，坚持深化市场化改革、扩大高水平开放，以新旧动能转换重大工程为引领，统筹推进稳增长、促改革、调结构、惠民生、防风险、保稳定工作，进一步稳就业、稳金融、稳外贸、稳外资、稳投资、稳预期，加快协同发展示范区建设，保持经济社会持续健康发展，以优异成绩庆祝中华人民共和国成立70周年。

主要预期目标是：地区生产总值增长6.5%左右，一般公共预算收入增长6%左右，固定资产投资增长6.5%左右，社会消费品零售总额增长8%左右，外贸进出口总额稳步增长，城镇和农村居民人均可支配收入分别增长7%和8%左右，居民消费价格涨幅控制在3%左右。

重点做好六方面的工作：

（一）加快新旧动能转换，做大做强实体经济

狠抓重大项目建设。坚持把大抓项目作为推动新旧动能转换的总抓手。高度重视项目储备工作，围绕创新驱动、结构调整、节能环保、乡村振兴、民生改善、重大基础设施等重点领域，实施新旧动能转换重大项目“515”工程，始终保持500个以上项目库存量，筛选100个项目重点调度，盯住50个项目快速推进。坚持“亩均论英雄”，把好项目质量关，提高资源利用和投入产出效率。建立项目高效落地保障机制，实行重大招商项目市县联动专班制度，从洽谈推进、签约落地、开工建设到投产达产全程紧密对接、跟进服务。对6个省重点项目和111个市重点项目实行市级领导干部包项目责任制，高效推进、高效落实，真正干出大上项目、快上项目的“德州速度”。

高质量开展“双招双引”。坚持把“双招双引”作为推动新旧动能转换的有力支撑，全市新增签约项目不少于210个，合同引进资金不低于800亿元。聚焦省“十强产业”和我市优势产业，搞好产业细分研究，做到产业精准、领域精准、政策精准、招商精准、投资精准，围绕集成电路、新能源汽车、现代高效农业等领域，尽快引进一批建链、补链、延链、强链的重点项目和技术团队，推动主导产业集群式发展。全面构建“部门条块结合、市县上下联动、产业前后衔接”的精准招商工作体系，聚焦欧美、日韩、“一带一路”沿线国家和京津冀、长三角、珠三角等国内重点区域，积极开展产业链定向招商、清单式目标招商、小团组靶向招商。精心组织举办高层次经济合作与招商推介活动，提升京津冀鲁资本技术交易大会等区域性展会的影响力。充分发挥鲁北人才改革试验区和招才引智“黄金30条”政策优势，聚焦产业需求，依托产业园区、创新平台、科研院所、龙头企业，引进领军人才、“双创”人才，强力构筑人才高地，促进德州加速崛起。

加快产业转型升级。持续做好淘汰落后产能、改造提升传统产业、培育壮大新兴产业三篇文章。综合运用能耗、环保、安全、质量等倒逼机制，大力淘汰落后产能，加快处置“僵尸企业”，释放发展空间。深度调整产业结构，聚焦化工、纺织服装、农副产品深加工、装备制造等传统优势产业，支持企业围绕产业链关键领域、薄弱环节、基础共性问题开展技术改造，着力抓好150个工业重点技改项目。鼓励企业加快新技术、新材料、新工艺、新装备推广使用，积极发展智能制造，推动一批智能工厂、智能车间改造，提升制造业整体水平。加快中小企业服务云平台建设，推动“企业上云”。支持具有关键核心技术、自主品牌的优势企业发展壮大，培育更多单项冠军、专精特新、隐形冠军、瞪羚企业。大力培育高端装备制造、生物技术、新能源、新材料、医养健康、现代农业等新兴产业，推进先进制造业与现代服务业深度融合。

深入推进创新驱动发展。围绕建设京津冀科技成果转化基地，充分发挥德州经济技术开发区“创新谷”、高新区（禹城）中央创新区、齐河中关村海淀高新园区等创新平台作用，大力促进科技成果转化。加快市应用技术研究院建设，构建开放、协同、高效的共性技术研发平台。鼓励企业加大研发投入，年内争取新认定省级高新技术企业50家以上，大中型工业企业研发平台占比达到80%以上。大力推动产学研合作，力争全市50%以上的规模以上企业与大院大所建立合作关系。加快推进清华大学山东化工产业技术研究院、中关村智能制造创新中心、中北大学研究所等研发机构落地见效。继续大力推进质量品牌建设，积极争创国家、省质量奖，提升“德州品牌”的整体竞争优势。

（二）坚持“两个毫不动摇”，发展壮大民营经济

着力营造良好发展环境。坚决落实省支持民营经济高质量发展35条、市支持民营经济高质量发展25条政策措施，让民营经济的各项政策在德州大地上落地生根、开花结果。牢固树立“政府创造环境、企业创造财富”理念，持续优化公共服务，建立企业纾困机制，以更加有力有效的政策举措，急企业之急、帮企业之困、解企业之难，让民营经济有肥沃的“成长土壤”、充足的“阳光雨露”。组织开展“金融机构走进民营企业”活动，建立银企对接长效机制，无还本续贷覆盖面扩大到2000家，千方百计缓解企业融资难、融资贵问题。积极构建“亲”“清”新型政商关系，常态化开展领导干部“进企业、解难题、促发展”工作，真正把民营企业家当作自家人，把民营企业的事当作自己事，将民营企业的难点痛点堵点作为工作的切入点，做到有求必应、无需不扰，“亲”而又“清”、“清”而又“亲”，多说“我来办”，少说“不好办”，以优质服务使德州的营商环境“更有温度”。

推动民营企业转型发展。继续实施小微企业治理结构和产业结构“双升”战略，推动“个转企、小升规、规改股、股上市”工作，支持优势企业开展形式多样的股权合作、产能合作、营销合作、技术合作，支持民营企业参与军民融合发展。推进民营企业融入新旧动能转换工程，实施特色产业提升计划，引导企业由低端产业进入先进制造业、现代服务业。实施“专精特新”“育苗扶壮”工程，支持优势小微企业通过技术创新向高端化发展。引导民营企业强化品牌意识，培育自主品牌，提升品牌效应。倡导现代企业家精神，鼓励企业在政策法规范围内放开手脚、大胆创业。深入实施企业家素质提升工程，支持企业参加全省民企接班人三年行动计划和企业家发展领航计划，继续组织企业骨干人员高端专题培训，培养具有现代经营理念和国际开放视野的企业家队伍。

（三）大力实施乡村振兴战略，努力在打造齐鲁样板中率先突破

积极推进产业振兴。深化农业供给侧结构性改革，加快农业由增产导向转向提质导向，提升农业发展质量、规模和效益，实现农业由大到强的战略性转变。推动藏粮于地、藏粮于技，夯实水利基础保障，抓好粮食生产功能区建设。调整传统种植结构，推进特色鲜明、优势集聚、市场竞争力强的农产品优势区建设。加快发展农业“新六产”，培育经营性和服务性合作组织，延长农业产业链，让更多的农产品就地转化、就地增值。积极推进现代农业产业园区和田园综合体建设，新建市级现代农业产业园10个以上，新（扩）建高标准蔬菜园区10个，打造万亩蔬菜功能保护区38个，新建和提升规模化标准化养殖场（区）200处以上。持续推进“放心农场”建设，坚持质量兴农，推行标准化生产，培育农产品品牌，全市“三品一标”产地认定面积稳定在60%以上。深化农村土地制度改革，稳步推进农村集体产权制度改革，探索农村土地“三权分置”有效形式。加快培育新型经营主体，发展多种形式农业适度规模经营，努力构建“龙头企业+合作社+基地+农户”发展体系，推进小农户和现代农业的有机衔接。

积极推进人才振兴。实施乡土人才选拔培养工程、新型职业农民培育工程和科技特派员行动计划，培养造就一支懂农业、爱农村、爱农民的“三农”工作队伍。搞好校地科技合作，借助农业院校、科研单位专家人才为乡村振兴提供智力支撑。创造良好环境，搭建创业平台，鼓励人才回乡、智力回乡、创业回乡，让各类人才在乡村各展其才、大显身手。

积极推进文化振兴。以社会主义核心价值观为引领，加强农村思想道德建设，实施“四德工程”，建好用好新时代文明实践中心，推进移风易俗，弘扬现代文明。实施“乡村记忆工程”，深入挖掘传统民俗、乡土文化，发展乡村特色文化旅游产业。加强乡村公共文化服务阵地建设，开展丰富多彩的群众文化活动。

积极推进生态振兴。高质量做好村庄发展规划编制，到2020年实现市域全覆盖。高标准实施9个省级美丽村居试点村建设，以点带面，打造齐鲁样板鲁西北风貌区。扎实开展农村人居环境整治三年行动，抓好“四好农村路”、电网升级改造、垃圾污水处理等公共基础设施建设，深入推进农村“七改”工程，健全完善“建、管、护”长效机制，让我们的农村环境越来越整洁、农村面貌越来越美丽、农民生活越来越幸福。

积极推进组织振兴。构建以党支部为核心的农村组织体系，健全自治、法治、德治相结合的治理模式，完善“四议两公开”决策机制、村民自治机制、民主监督机制。深化拓展“强村固基”工程，大力推行农村党支部领办创办土地股份合作社，发展壮大农村集体经济。创新农村社会治理，深化城乡网格化服务管理，打造党组织领导下的共建共治共享的乡村治理格局。加强村级小微权力监督制约，从源头上遏制群众身边的“微腐败”。

巩固提升脱贫成果。切实扛起脱贫攻坚政治责任，尽锐出战，精准发力，坚决打好巩固提升年度战役。拓宽贫困户增收渠道，全面提升产业扶贫质量和效益。严格落实保障性扶贫兜底政策，完善“大救助”体系，深化教育扶贫、健康扶贫，加快危房改造，巩固“两不愁三保障”成效。开展扶贫扶志行动，提升贫困群众内生动力，增强贫困人口技能水平和就业能力，确保脱贫群众生活稳得住、逐步能致富，在全面建成小康社会的道路上，一个都不能少，一个都不能掉队。全面提升贫困村整体发展水平，用好乡村振兴、美丽乡村建设和人居环境整治等政策，补齐贫困村短板弱项，加大沉睡资源利用力度，千方百计增加村集体收入。扎实做好精准扶贫“回头看”，对2016年以来的省定扶贫工作重点村、贫困户、贫困人口，进行一次大回访，及时发现问题、解决问题，确保扶贫工作务实、脱贫过程扎实、脱贫结果真实。

（四）全面深化改革、扩大开放，进一步提高发展活力和外向度

持续推进对内对外双向开放。坚定不移推进协同发展示范区建设，深度对接京津、服务雄安、融入省会，围绕“精准承接、科创协作、农业改革、人才引培、生态环保、资源互通”六条主线，组织开展对接交流活动，推动一批重大政策、重要事项和重点项目落地实施。继续做好扶贫协作和援疆工作。全面落实稳外贸、稳外资工作要求，保持外经外贸外资平稳健康发展。精准对接与我市产业契合度高的行业领军企业，加快推进与光大、华润、普洛斯的战略合作，培育一批有国际影响力的特色园区、高端产业基地、知名品牌。加快开发区体制机制创新，每个省级以上开发区聚力发展1至2个主导产业，努力打造一批百亿级产业集群。针对东盟、欧洲、中亚、日韩等重点区域，办好具有德州特色的重大经贸活动，全力支持企业“走出去”，拓展“一带一路”等新兴市场，让更多的“德州制造”“德州创造”走出国门、走向世界。侯卫东官场笔记

稳妥有序推进各领域改革。以“一次办好”改革为统领，深化“放管服”改革，持续打造精简高效的政务生态。更大力度推进市级简政放权，深化扩权强县改革，实施“市县同权”，凡是法律法规没有明确实施层级且基层有实际需求的，原则上交由县（市、区）实施。落实好国家出台的减税降费等各项措施，为实体经济尤其是民营企业、小微企业减负，增强市场主体活跃度。扎实推进“减证便民”“证照分离”、降低企业准入门槛等措施，加快政务信息系统整合共享，建立营商环境评价机制，有效降低制度性交易成本。加强中介服务市场培育，建立健全退出机制，全面提升服务质量。继续做好政府系统机构改革相关工作，有序推进乡镇行政管理体制改革。扎实推进市场监管、生态环境保护、文化市场、交通运输、农业等领域综合执法改革，推动城市管理等跨领域跨部门综合执法。完善财政管理体制，推进预算管理改革，全面实施预算绩效管理。依法合规开展第四次经济普查和第三次国土调查。认真做好校长职级制、教师“县管校聘”、中小学招生制度、“三医联动”、基层民政服务体系、司法体制、新闻出版、广播电视等改革事项，让改革发展成果更多更公平惠及全体人民。

（五）转变城市发展方式，全面提升城市品质

持续完善城市功能。优化城市设计，统筹建筑布局，协调城市景观，持续推进“城市双修”。组织实施5大类65个公共城建项目，集中实施道路建设、雨污分流、棚户区改造等项目。加快推进地下综合管廊和海绵城市建设，继续实施一批污水垃圾处理、供排水、燃气、热力等基础设施工程。坚持重点片区有序更新和微改造协同并进，启动新一轮老旧小区综合整治，完善治安防控、环卫消防、水电气暖等硬件设施，建立物业管理长效机制，让群众居住环境更加整洁宜居。继续加快东部城区开发建设，引进落地一批具有产业引领和支撑作用的大项目、好项目，更好地发挥城市发展增长极作用。超前谋划5G网络、人工智能、工业互联网、物联网等新型基础设施。高标准推进京台高速德州至齐河段改扩建等重点工程建设，积极推进德高（高青）高速德州段、德单（单县）高速德州段、济南二环线西环项目前期工作，积极争取纳入京津冀一小时交通圈城际铁路建设，为全省对接京津冀协同发展打开新通道。

提升城市治理水平。巩固国家卫生城市复审成果，健全长效机制，提高精细化管理水平。推进绿化美化亮化，着力治脏、治乱、治差、治堵，增加绿地、游园、停车场，推进城市“微更新”，优化“微服务”，不断提升人居环境质量。加快智慧城市建设，推动城市管理向个性化、精细化、数字化、智能化方向转变。加强和完善城市运行安全保障体系，全面提高公共安全水平。认真做好全国文明城市创建工作，提高市民文明素质，提升城市文明程度。统筹考虑群众工作、生活、就学等需要，按照居民生活半径，优化配建一批教育医疗、文化体育、社区养老、便民市场、公共厕所等设施，让群众通过身边的小事体会到城市的温度。

（六）持续保障改善民生，切实增强人民群众获得感幸福感安全感

着力改善生态环境。解决好人民群众反映强烈的突出环境问题，是改善环境民生的迫切需要。坚持以生态优先、绿色发展为导向，把德州作为京津南部、山东北部重要生态功能区规划、建设和管理，全面落实“1+1+8”系列方案，积极开展“四减四增”三年行动，坚决打好八场标志性战役。把打赢蓝天保卫战作为重中之重，以空气质量明显改善为刚性要求，坚守阵地、巩固成果，以钉钉子精神治理大气污染。实施水污染防治行动计划，加快补齐城乡污水收集和处理短板。推进水生态修复工程建设，加强饮用水水源地保护，保障饮水安全。推进城市节水工作，开展地下水超采区综合整治，力争通过城市节水一级评价标准。推进生活垃圾无害化处理设施建设，提升城乡垃圾处理一体化水平。落实土壤污染防治行动计划，强化土壤污染管控和修复，加强危险废弃物处置监管。做好中央、省环保督察反馈问题整改。注重区别对待、精准施策，强化服务，帮助企业制定环境治理解决方案，协同推动经济发展和环境保护，实现共促共进。

推动教育高质量发展。启动国家义务教育优质均衡发展县（市、区）创建，编制德州教育现代化相关规划和实施方案。大力推动城镇新建居住小区按规定配建相应教育设施，建立解决大班额工作长效机制，为2020年全面消除大班额夯实基础。深入实施学前教育三年行动计划，新建、改扩建普惠制幼儿园133所，新增学位2万个。加大高中学校建设力度，尽快扩充高中学位，努力满足人民群众对优质高中教育的需求。大力发展现代职业教育，推进公办中等职业学校规范化建设，争取国家级中职技能大赛项目长期落户德州，支持德州职业技术学院创建山东省优质高职院校。加强教师队伍建设，开展乡村教师公寓建设试点。建立校外培训机构清理整治长效机制，切实减轻学生课业负担。广泛开展全民健身活动，积极筹备德州市第六届运动会。

加快发展卫生健康事业。抓好3个市级和15个县级重点医疗卫生项目建设，优化中心城区医疗资源布局，提升县域医疗服务能力，完成基层医疗卫生机构标准化建设。实施重点专科培育计划和学科带头人、技术骨干培养计划，培育30个临床（中医）重点专科，培养50个学科带头人和技术骨干。实施优质医疗资源下沉计划，做好城乡医院对口支援，选派医务工作者到驻点乡镇卫生院开展帮扶。加强紧密型医疗共同体建设，实现各县（市、区）全覆盖。完善公共卫生服务体系，做好职业病、慢性病、传染病和重大疾病防治，提升卫生应急能力。强化中医药传承创新，加强中医药设备配置和人员配备，促进中医药事业振兴发展。全面提高出生人口素质，实施好“母婴安全行动计划”“健康儿童行动计划”，全市孕前、产前、新生儿筛查率分别达到85%、80%、98%以上。大力发展医养结合和医养健康产业，加强残疾人康复服务，建立残疾儿童康复救助制度。深入开展爱国卫生运动，提升全民健康素养。

完善社会保障体系。把稳就业摆在突出位置，实施就业优先政策，强化创业带动就业，统筹抓好高校毕业生、农民工、城镇困难人员等各类群体就业，全年城镇新增就业6.1万人。全面实施全民参保计划，加快社会保障法定人群全覆盖进程。完善基本养老保险制度，提升统筹层次，稳步提高待遇保障水平。建立职工长期护理保险制度。加强社保基金监管，确保基金安全。抓好农民工工资发放工作，促进劳动关系和谐稳定。做好退役军人安置、管理和保障工作。 高度重视养老事业，推进社区居家和机构养老服务发展，全市新增养老床位3000张，新建城市日间照料中心10处、农村互助幸福院40处。每个县（市、区）至少建成1处社会福利中心，实行特困人员中失能、半失能人员县级集中供养。继续做好低保、特困提标工作，建立乡镇临时救助金制度，开辟市县乡村临时救助绿色通道。健全房地产市场健康发展长效机制，完善住房市场体系和住房保障体系。

繁荣发展文化和旅游事业。完善市县乡村四级公共文化服务阵地，推进基层综合性文化服务中心建设，提升公共文化场馆服务效能。传承发展优秀传统文化，推出一批具有地方特色的精品力作，办好“一村一年一场戏”等系列文化惠民活动。加强文化遗产保护传承，推动大运河文化带建设，延续历史文脉，留住城市记忆。积极推动文化旅游产业融合发展，实施重点项目带动，支持齐河放大泉城欧乐堡、大地自然博物馆等项目群的产业优势创建黄河国际生态城国家级旅游度假区，支持夏津黄河故道生态旅游区等创建省级旅游度假区。大力发展全域旅游，加快景区宾馆饭店、娱乐购物等旅游设施提档升级，支持齐河、夏津、庆云、乐陵创建省级全域旅游示范区。扎实推动乡村旅游纵深发展，支持10个特色小镇打造省级精品旅游小镇，鼓励引导依托水系、湿地、景区发展一批乡村旅游集群片区和旅游特色园区。

着力防范化解重大风险。坚持底线思维，增强忧患意识，提高防控能力，坚决打好防范化解重大风险攻坚战。认真落实中央稳金融相关要求，坚守防范系统性金融风险的工作底线。建立大数据监管平台和企业信用体系，健全金融风险监测防控体系，强化对金融风险的监测预警和及早处置。指导债权银行建立银行债权人委员会，加大不良贷款处置力度，做好重点企业、法人机构风险化解。持续开展严厉打击恶意逃废金融债务专项行动，进一步优化金融生态环境。加强政府债务管理，防范化解隐性债务风险。严格落实安全生产责任制，推进安全生产风险分级管控和隐患排查治理双重预防体系建设，强化应急管理基层基础工作。着力抓好重点行业领域专项整治，确保全市安全生产形势持续稳定。深化平安德州建设，持续实施“雪亮工程”，创新完善立体化、信息化社会治安防控体系，深入推进扫黑除恶专项斗争，严厉打击黑拐骗、黄赌毒等违法犯罪活动。组织开展矛盾纠纷大排查、大化解，进一步畅通信访渠道，打造“阳光信访”。扎实推进公共法律服务体系建设，提升法律援助和普法依法治理水平。深入推进“食安德州”建设，确保广大群众“舌尖上的安全”。

三、加强政府自身建设，狠抓工作落实

全面贯彻省、市“担当作为、狠抓落实”工作动员大会精神，以深入开展“工作落实年”为总抓手，心无旁骛干事业，聚精会神抓落实，勇做新时代泰山“挑山工”。

（一）提高政治站位，忠诚履职抓落实。坚持把党的政治建设放在首位，树牢“四个意识”，坚定“四个自信”，做到“两个维护”。自觉用习近平新时代中国特色社会主义思想武装头脑、指导实践、推动工作，在思想上政治上行动上同以习近平同志为核心的党中央保持高度一致，确保党中央国务院、省委省政府和市委决策部署落地见效。始终坚持对党绝对忠诚，牢牢把握正确政治方向，把严守政治纪律和政治规矩体现在具体行动上、落实到政府工作中。深入开展“不忘初心、牢记使命”主题教育。严格落实意识形态工作责任制，守牢意识形态阵地。

（二）坚持依法行政，科学决策抓落实。遵守宪法法律，建设法治政府，运用法治思维和法治方式解决问题、推动发展。坚持依法科学决策，严格履行决策法定程序，健全重大行政决策公众参与、专家论证、风险评估、合法性审查制度。依法接受人大及其常委会的监督，自觉接受人民政协的民主监督，主动接受社会和舆论监督，让权力在阳光下运行。推进“开门决策”制度化，全面贯彻以人民为中心的发展思想，更加注重各项工作的群众感受，把评判权交给服务对象和基层群众。全面推进政务公开，加强政府信用建设，着力提升政府公信力。

（三）聚焦重点难点，扑下身子抓落实。坚持问题导向、目标导向、结果导向和实绩评价相统一，全力抓重点、破难点、补短板，在抓贯彻、抓执行、抓推进、抓见效上狠下功夫，推动各项工作早见成果、多见成效。出台的政策、定下的任务、承诺的事情，盯住不放、一抓到底。始终把基层一线作为抓落实的主阵地，坚持以上率下，当好“施工队长”，推动问题在一线发现、政策在一线实施、工作在一线推进。建立重点工程、重大项目、重要事项责任清单，组建工作专班，实施专项推进，以目标倒逼责任，以时限倒逼进度，变“规划图”为“施工图”，变“时间表”为“计程表”。坚持严管和厚爱、激励和约束并重，健全完善正向激励机制和容错纠错机制，旗帜鲜明为敢于担当者担当、为敢于负责者负责，对庸政懒政怠政、不作为、慢作为、乱作为严肃问责。大力精简会议文件，统筹规范督查检查考核，减轻基层负担，关心关爱基层干部，让基层腾出更多精力抓落实。

（四）恪守清正廉洁，严明纪律抓落实。认真落实全面从严治党要求，切实履行“一岗双责”，以永远在路上的坚韧和执着，持续强化政府系统党的建设和廉政建设。坚持公正用权、谨慎用权、依法用权，主动接受法律监督、监察监督和人民监督，加强公共资金、公共资源交易、国有资产、自然资源等领域管理和审计监督，形成靠制度管权、管事、管人的长效机制。锲而不舍落实中央八项规定及其实施细则精神，驰而不息纠正“四风”，坚决破除形式主义、官僚主义。坚决把纪律和规矩挺在前面，打造忠诚干净担当的干部队伍，推动政府各项工作高效有序落实。

各位代表，新时代是奋斗者的时代，更是追梦人的舞台。让我们更加紧密地团结在以习近平同志为核心的党中央周围，高举习近平新时代中国特色社会主义思想伟大旗帜，在市委的坚强领导下，不忘初心、牢记使命，担当作为、狠抓落实，加快建设协同发展示范区，为决胜全面建成小康社会作出更大贡献，以优异成绩迎接新中国成立70周年！
